# Editorial: Insights in pediatric Endocrinology: 2021

**DOI:** 10.3389/fendo.2022.977912

**Published:** 2022-07-25

**Authors:** Sally Radovick, Madhusmita Misra

**Affiliations:** ^1^Department of Pediatrics, Rutgers, Robert Wood Johnson Medical School, New Brunswick, NJ, United States; ^2^Division of Pediatric Endocrinology, Massachusetts General Hospital, Boston, MA, United States

**Keywords:** precocious puberty, GnRH agonist, pituitary, estrogen replacement therapy, Turner syndrome, adrenal insufficiency

This special edition Research Topic was designed to include novel and upcoming research spanning various pediatric endocrine topics, to discuss current challenges and provide new insights and perspectives to guide the field into the future.

Three papers on this topic are related to pubertal issues, and report on (i) diagnostic testing for central precocious puberty (CPP), (ii) the induction of puberty in hypogonadal girls with Turner syndrome, and (iii) a possible side effect of management of CPP with gonadotropin releasing hormone agonists.

Cao et al. performed a retrospective analysis of GnRH agonist stimulation testing in 1492 girls diagnosed with precocious puberty, of whom 518 had CPP. The study demonstrated that a basal LH value of >0.535 mIU/mL could be used to diagnose CPP without the need for a GnRH agonist stimulation test. Further, they determined that a single 60-minute post GnRH agonist LH concentration of >7.65 mIU/mL or LH/FSH ratio >0.603 was sufficient to diagnose CPP without the need for a full 3-hour GnRH agonist stimulation test. These are clinically relevant findings with the potential to reduce the duration of the GnRH agonist test.

Obara-Moszynska et al. retrospectively examined the impact of estrogen replacement therapy (ERT) using 17-β estradiol (transdermal or oral) in 40 girls with Turner syndrome on uterine volume using ultrasound, compared to 20 healthy controls who underwent spontaneous puberty. A progestin, dydrogesterone, was added after two years of ERT or after the first menstrual period. While uterine volume before starting ERT did not differ in girls with TS vs. prepubertal controls, uterine volume was lower by 14.6 ml in patients with TS at the conclusion of puberty induction (≥ 36 months of ERT or ≥ 12 months after menarche) compared to postpubertal controls. Uterine volume at the end of the assessment period was not related to chronological, menarchal, or bone age, FSH concentration, the estradiol dose at the last visit, the time between estrogen induction and menarche, body size, uterine volume at baseline, or early changes in uterine volume with ERT. However, the study did not look at cumulative estrogen exposure in this context, which may have provided insight into this finding. In contrast to uterine volume, fundocervical anterior to posterior ratio (FCR) did not differ between girls with TS before and after puberty induction compared to prepubertal and postpubertal controls, respectively. Thus, the volume of the uterus following puberty induction appears to be significantly smaller than in healthy girls who undergo spontaneous puberty and raises concerns regarding adherence to the prescribed regimen, whether or not the dose of ERT is sufficient to enable full uterine maturation, whether a longer duration of follow-up will demonstrate a catch-up in uterine volume, and whether insufficient estrogen exposure during ERT has implications for other outcomes, including bone outcomes. Given that the final dose of transdermal estradiol in girls with Turner syndrome was 12.5-50 mcg per day, it does seem that the dose of estradiol used for puberty induction may have been insufficient in a subset of study participants.

Leite et al. tackled the concern that GnRH agonist treatment for CPP is associated with weight gain. In an observational retrospective study of 92 children of both sexes (8% boys) being treated for CPP, they noted that BMI-SDS increased during treatment among girls (but not boys) and decreased a year after stopping GnRH agonist therapy. The gain in BMI-SDS during treatment was associated with baseline BMI-SDS such that normal-weight girls, but not those with overweight or obesity, had a greater risk of gaining weight during treatment. The mechanism underlying this effect is unclear. If these results are confirmed in additional studies, parents of children receiving GnRH agonist therapy (particularly for normal-weight girls) should be cautioned about this effect, while reassuring them that the effect reverses after treatment is complete.

The other three manuscripts focus on a variety of studies in pediatric endocrinology. These include (i) an investigation into the relationship between obstructive sleep apnea (OSA), obesity, and activation of the hypothalamic-pituitary-adrenal (HPA) axis, (ii) the management of adrenal insufficiency (AI) by parents, and (iii) a rare case of duplication of the pituitary gland (DPG)-plus syndrome presenting with precocious puberty.

Berdina et al. sought to determine whether obstructive sleep apnea (OSA) and obesity are associated with stress system activation involving the hypothalamic-pituitary-adrenal (HPA) axis in childhood and adolescence, as they are in adults. They examined diurnal salivary cortisol as a measurement of HPA axis function in adolescents with obesity with and without OSA, and the relationships between cortisol levels, body weight, and parameters of polysomnography (PSG). The results of their study provided evidence for alterations in diurnal cortisol production in adolescents with obesity, which may indicate a chronically stressed HPA axis. There were significant differences in salivary cortisol parameters between participants with and without OSA. Furthermore, OSA patients had more associations between time-point cortisol levels and OSA-related indices.

Worth et al. call for training programs for parents of children with adrenal insufficiency (AI) in the management of adrenal crisis (AC) to encourage the use of parenteral hydrocortisone. This recommendation is based on a 3-pronged study to determine what parents do at home to manage symptomatic AI. All patients received an increase in oral hydrocortisone before admission, but only two received intramuscular hydrocortisone. Questionnaires revealed that 79% of parents reported confidence in administering intramuscular hydrocortisone, and only 20% identified a missed opportunity for injection. They concluded that in children experiencing AC, parents followed ‘sick day’ guidance for oral hydrocortisone but rarely administered intramuscular hydrocortisone. Hence, they recommended programs for parents to enhance learning focused on the use of parenteral hydrocortisone for their children with AC.

Prezioso et al. present a case report of duplication of the pituitary gland (DPG)-plus syndrome, a rare developmental disorder characterized by multiple midline and central nervous system malformations, which may be clinically associated with endocrine abnormalities. The manuscript describes a 5.9-year-old female child with evidence of advancing puberty. DPG-plus syndrome was diagnosed after birth, when she appeared small for gestational age with lingual hypoplasia, cleft palate, right choanal stenosis, nasopharyngeal teratoma, and facial dysmorphisms associated with paired infundibula and pituitary glands. Precocious puberty was diagnosed and hormonal suppression treatment was started. An array-CGH revealed a 2p12 deletion, although it was not associated with the syndrome. This case demonstrates that DPG-plus syndrome must be considered in the presence of midline and craniofacial malformations. Endocrinological evaluations should be performed for the prompt and appropriate management of pubertal anomalies.

We commend the authors for their work and their contributions and look forward to submissions to Insights in Pediatric Endocrinology, 2022.

## Author contributions

All authors listed have made a substantial, direct, and intellectual contribution to the work and approved it for publication.

## Funding

SR is funded by R01HD068777, R01HD041702, R01HD086013, 1R61HD105619, U01HD086838, 1KL2TR003018 and MM is funded by NIH grants R01 DK103946, UL1T R002541, R01MH116205, R01DK122581 and R01DK124223. All grants are from the National Institutes of Health.

## Conflict of interest

Author SR serves as a consultant for CVS/Caremark. Author MM has served on the scientific advisory board of Ipsen and Abbvie and as a consultant for Abbvie and Sanofi.

## Publisher’s note

All claims expressed in this article are solely those of the authors and do not necessarily represent those of their affiliated organizations, or those of the publisher, the editors and the reviewers. Any product that may be evaluated in this article, or claim that may be made by its manufacturer, is not guaranteed or endorsed by the publisher.

